# Study on Broadband and High-Performance Microwave-Absorbing Spinel NiCo_2_O_4_ Regulated by Fe Doping

**DOI:** 10.3390/nano16130806

**Published:** 2026-06-30

**Authors:** Yuanyuan Lv, Yujia Liu, Danyang Bai, Neng Li, Jin Liu

**Affiliations:** School of Communication and Information Engineering, Xi’an University of Science and Technology, Xi’an 710054, China

**Keywords:** Fe doping, NiCo_2_O_4_, electromagnetic wave absorption, impedance matching, synergistic loss

## Abstract

Spinel NiCo_2_O_4_ has emerged as a promising microwave absorption material due to its unique crystal structure and abundant defect sites. Nevertheless, its low intrinsic electrical conductivity leads to insufficient conductive loss and unsatisfactory high-frequency impedance matching, severely limiting the simultaneous realization of strong electromagnetic attenuation and broad absorption bandwidth. Fe^3+^ doping is an effective modification strategy for NiCo_2_O_4_ by virtue of its matched ionic radius and dual modulation capability for dielectric and magnetic properties. Herein, pristine and Fe-doped NiCo_2_O_4_ absorbers with different doping contents (4%, 6%, 8%) were fabricated via a hydrothermal–calcination route, and the correlation between Fe doping concentration, microstructure, electronic structure, electromagnetic properties, and microwave absorption performance was systematically investigated. Benefiting from moderate 6% Fe doping, the optimized F6 sample exhibits a refined porous nano-agglomerate structure, which provides abundant heterogeneous interfaces and pore channels for electromagnetic wave scattering and attenuation. The introduced oxygen vacancies and balanced Ni^2+^/Ni^3+^, Co^2+^/Co^3+^, and Fe^2+^/Fe^3+^ mixed-valence states effectively strengthen interfacial and dipole polarization, while the optimized electrical conductivity and magnetic properties synergistically boost conductive and magnetic losses. Owing to the dual-loss synergism and superior impedance matching (58% proportion of Δ < 0.4), the F6 sample achieves an excellent minimum reflection loss of −62.7 dB at 2.2 mm and a wide effective absorption bandwidth of 4.6 GHz. This work clarifies the intrinsic structure–performance mechanism of Fe-doped NiCo_2_O_4_, providing a reliable and feasible strategy for the design and preparation of high-performance spinel-type microwave-absorbing materials.

## 1. Introduction

With the rapid development of fifth-generation mobile communication (5G), the Internet of Things, radar detection, and highly integrated electronic devices, electromagnetic waves are playing an increasingly important role in human production and daily life. However, the ubiquity of electromagnetic waves has also brought about increasingly severe electromagnetic pollution [1,2]. Electromagnetic interference not only seriously affects the normal operation of precision electronic equipment but also poses potential threats to human health through long-term exposure. It has become the fourth largest environmental pollutant after air pollution, water pollution, and noise pollution [3,4]. Meanwhile, in the military defense field, the continuous advancement of radar detection technology imposes higher demands on the radar stealth capability of weaponry such as aircraft. Against this background, the development of high-performance electromagnetic wave-absorbing materials that combine the characteristics of “thinness, lightweight, wide bandwidth, and strong absorption” has become one of the central research directions in the interdisciplinary field of materials science and electronic engineering [5,6,7,8].

An ideal absorbing material should satisfy two core requirements: impedance matching and strong attenuation. Impedance matching ensures that electromagnetic waves can enter the material to the greatest extent, rather than being reflected at the surface, while strong attenuation guarantees that the energy of the electromagnetic waves entering the material can be effectively dissipated into heat or other forms of energy through various mechanisms, such as dielectric loss and magnetic loss [9,10]. Based on these two design principles, researchers have developed a variety of absorbing material systems, including ferrites, metal powders, conductive polymers, carbon-based materials (carbon nanotubes, graphene, biomass carbon), and transition metal compounds [11]. However, traditional absorbing materials often face their own performance bottlenecks: ferrites and metal powders have high density, narrow bandwidth, and poor environmental stability; carbon-based materials, although lightweight, are difficult to tune in terms of impedance matching; and single dielectric or magnetic materials can hardly achieve both wide bandwidth and strong absorption simultaneously [12,13]. In recent years, transition metal oxides have gradually become a research hotspot in the field of absorbing materials due to their tunable structure, good thermal stability, and coexistence of multiple loss mechanisms [14,15].

Among the numerous transition metal oxides, nickel cobaltite (NiCo_2_O_4_) has attracted considerable attention due to its unique crystal structure and excellent physicochemical properties. NiCo_2_O_4_ possesses an inverse spinel structure, in which Ni^2+^ ions occupy octahedral sites while Co^3+^ ions occupy both tetrahedral and octahedral sites. This distinctive cation distribution endows the material with abundant electron transition pathways and a relatively high intrinsic electrical conductivity [16,17]. Moreover, NiCo_2_O_4_ contains two transition metal elements, Ni and Co, allowing synergistic optimization of dielectric and magnetic properties by tuning the Ni/Co ratio. Previous studies have demonstrated that NiCo_2_O_4_ exhibits significant potential for electromagnetic wave absorption. Through morphology engineering (e.g., multi-shell hollow spheres, nanosheets, nanoflowers) and defect engineering (e.g., regulation of oxygen vacancies and lattice defects), its dielectric loss capability can be effectively enhanced [18]. A research team from Northwestern Polytechnical University systematically elucidated for the first time the polarization loss mechanism induced by lattice defects in NiCo_2_O_4_, revealing that NiCo_2_O_4_, owing to its abundant defect sites in the spinel structure, shows superior absorbing performance compared to other cobalt-based spinel materials. This work highlighted the critical role of defect engineering in modulating the dielectric loss capacity of materials and laid a theoretical foundation for the rational design of NiCo_2_O_4_-based absorbing materials. Liu et al. synthesized core–shell structured nanorod composites composed of Fe_3_O_4_/SiO_2_/graphene using a facile wet-chemical method, achieving a minimum reflection loss of −27.1 dB at 12.2 GHz with a coating thickness of only 1.5 mm [19]. Yang et al. prepared α-Fe_2_O_3_ microplates with a thickness of 5 mm and a minimum reflection loss of −41.67 dB [20]. Nevertheless, despite the remarkable progress achieved by NiCo_2_O_4_ in the field of wave absorption, the issue of its relatively low intrinsic electrical conductivity still restricts further improvement in absorbing performance, especially the synergistic optimization of strong absorption and wide bandwidth in the high-frequency range.

To address the above-mentioned issues, element doping has proven to be an effective strategy for modulating the electromagnetic parameters and loss mechanisms of materials. By introducing heteroatoms into the crystal lattice, the electronic structure, carrier concentration, and defect state density of the material can be altered, thereby enabling the regulation of the permittivity, electrical conductivity, and polarization loss capability. Regarding the choice of dopant, iron (Fe) offers unique advantages because of its combined dielectric-tuning and magnetic properties [21]. The ionic radius of Fe^3+^ is close to those of Ni^2+^ and Co^3+^, allowing Fe^3+^ to partially substitute Ni or Co sites in the lattice while maintaining the integrity of the spinel structure. More importantly, the introduction of Fe can simultaneously affect the dielectric and magnetic properties of the material. On the one hand, Fe doping introduces additional defects and charge imbalance into the lattice, enhancing dipole polarization and interfacial polarization. On the other hand, the magnetic characteristics of Fe help to improve the magnetic loss capability, achieving a synergistic enhancement of dielectric and magnetic losses [22]. This synergy of multiple loss mechanisms is expected to break through the performance bottleneck of a single loss mechanism, providing an effective route to achieve wide-bandwidth and strong absorption. Previous studies have shown that Fe doping can significantly improve the electromagnetic wave absorption performance of other spinel-type oxides (e.g., Sc_2_Si_2_O_7_, SiOC, etc.).

Nevertheless, systematic research on the microwave absorption performance of Fe-doped NiCo_2_O_4_ remains relatively limited. Very recently, Xu et al. reported the effect of Fe doping on the microwave absorption properties of NiCo_2_O_4_ spinel oxide, finding that Fe doping effectively increased the electrical conductivity of the material and thereby enhanced the contribution of conduction loss to electromagnetic wave attenuation. At an Fe doping level of 0.08, the sample achieved a maximum reflection loss of −48.90 dB at 15.40 GHz, with an effective absorption bandwidth of 4.20 GHz. This result demonstrates that the Fe doping strategy has significant potential for improving the absorption performance of NiCo_2_O_4_. However, to date, the structure–property relationship between doping concentration and absorption performance in the Fe-doped NiCo_2_O_4_ system has not been systematically elucidated. The underlying mechanisms by which Fe doping affects the defect structure, electrical conductivity, and dielectric relaxation behavior of NiCo_2_O_4_ still require further investigation, especially the synergistic contribution of conduction loss, polarization loss, and magnetic loss in the Fe-doped system.

Based on the above research background, this paper systematically investigates the effects of different Fe doping amounts on the microstructure, electromagnetic parameters, and microwave absorption performance of NiCo_2_O_4_ spinel oxide. By adjusting the Fe doping concentration, the evolution of the phase composition, morphology, dielectric properties, and magnetic properties of the materials is systematically analyzed. The enhancement mechanism of conduction loss induced by Fe doping and the contribution of dielectric–magnetic loss synergy to absorption performance are deeply revealed. This work aims to establish the structure–property relationship between Fe doping concentration and absorption performance, provide theoretical guidance for doping engineering strategies for high-performance spinel-based absorbing materials, and promote the practical application of NiCo_2_O_4_-based materials in the field of electromagnetic wave absorption.

## 2. Experiment

### 2.1. Synthesis of Fe-Doped NiCo_2_O_4_

Fe-doped NiCo_2_O_4_ powder materials were prepared via a two-step process combining hydrothermal synthesis and high-temperature annealing. The detailed procedures are as follows. First, 0.5 mmol of nickel nitrate hexahydrate (Ni(NO_3_)_2_·6H_2_O, Tianjin, China) and 1 mmol of cobalt nitrate hexahydrate (Co(NO_3_)_2_·6H_2_O, Shanghai, China) were dissolved in 70 mL of deionized water. Subsequently, iron(III) nitrate nonahydrate (Fe(NO_3_)_3_·9H_2_O, Tianjin, China) was separately introduced with Fe doping concentrations of 0 at.%, 4 at.%, 6 at.% and 8 at.%, where the concentration was defined relative to the total metal cation content. The mixture was magnetically stirred for 10 min until complete dissolution. Subsequently, 20 mmol of urea (CO(NH_2_)_2_) was added to the mixed solution, and stirring was continued for another 30 min to form a homogeneous precursor solution. The resulting precursor solution was transferred into a 100 mL Teflon-lined stainless steel autoclave, sealed, and placed in an oven at 120 °C for 10 h of hydrothermal reaction. After the reaction, the autoclave was naturally cooled to room temperature. The obtained precipitate was collected, washed three times with deionized water to remove residual impurities, and then dried in an oven at 70 °C. The dried precursor powder was placed in a muffle furnace, heated to 400 °C at a ramp rate of 5 °C/min, and calcined at this temperature for 2 h. After cooling to room temperature, the Fe-doped NiCo_2_O_4_ powder materials with different doping contents were obtained, which were denoted as F0, F4, F6, and F8, respectively.

### 2.2. Characterizations

The morphology and structure of the Fe-doped NiCo_2_O_4_ powder materials were characterized by X-ray diffraction (XRD, Shimadzu, Japan, 6100) and scanning electron microscopy (SEM, Germany, ZEISS Gemini SEM360). X-ray photoelectron spectroscopy (XPS, USA, Thermo Fisher-ESCALABXi) was employed to analyze the elemental composition and chemical states on the surface. The porosity and specific surface area of the samples were measured using a BET surface area analyzer (Japan, MicrotracBEL-BELSORP MAX II). Magnetic properties and electrical conductivity were characterized by a vibrating sample magnetometer (VSM, USA, Lake Shore-8604) and a resistivity testing system (FT-300I). The electromagnetic properties were analyzed using a vector network analyzer (VNA, China, CETC 1464-F). For electromagnetic measurements, the Fe-NiCo_2_O_4_ powder materials were mixed with paraffin wax at a mass ratio of 30:70, and the mixture was compressed into toroidal rings with an inner diameter of 3.00 mm, an outer diameter of 7.00 mm, and a thickness of 2.00 mm under a pressing pressure of 2.0 MPa.

## 3. Results and Discussion

**Figure 1:**
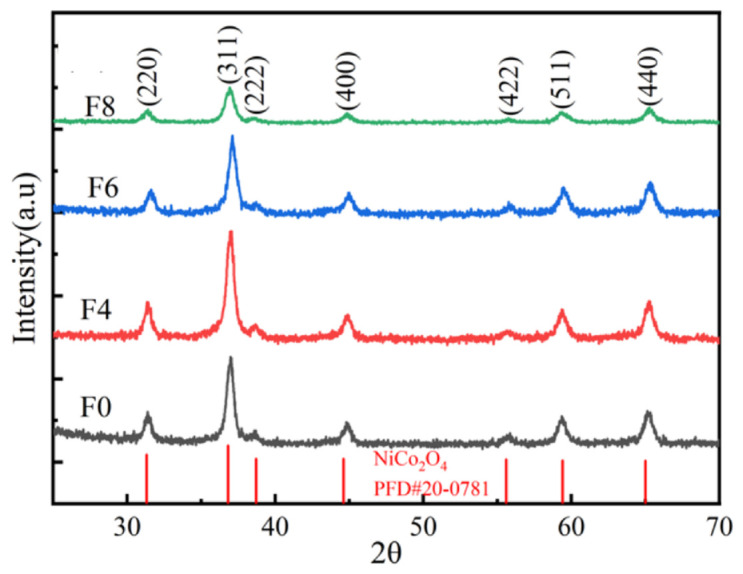
X-ray diffraction (XRD) patterns collected over a 2θ scanning range of 20–70° for pristine (F0) and Fe-doped NiCo_2_O_4_ powder materials (F4, F6, F8).

Figure 1 presents the X-ray diffraction (XRD) patterns of pristine (F0) and Fe-doped NiCo_2_O_4_ powder materials (F4, F6, F8). All samples exhibit distinct diffraction peaks at 2θ ≈ 31.1°, 36.7°, 38.3°, 44.6°, 55.4°, 59.1°, 64.9°, and 68.2°, which correspond to the (220), (311), (222), (400), (422), (511), (440), and (531) crystal planes of the spinel-structured NiCo_2_O_4_ (JCPDS #20-0781), respectively. These diffraction peaks are in good agreement with the standard card, indicating the successful synthesis of NiCo_2_O_4_ materials with a spinel structure. These sharp and intense diffraction peaks match well with the standard PDF pattern, verifying the successful fabrication of phase-pure spinel NiCo_2_O_4_. Furthermore, no characteristic peaks of impurities such as Fe_2_O_3_ or Fe_3_O_4_ are detected, indicating that Fe elements have been successfully incorporated into the crystal lattice without disrupting the spinel framework.

**Figure 2:**
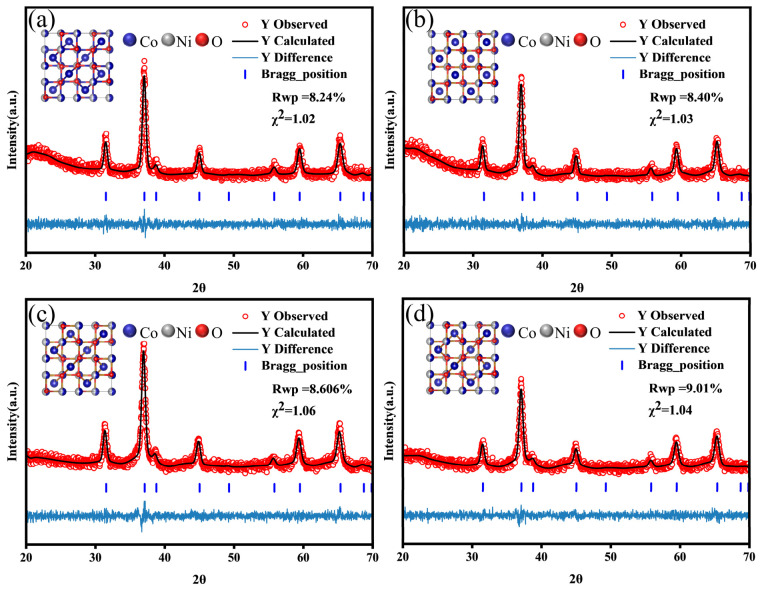
Refinement result of XRD pattern of pristine and Fe-doped NiCo_2_O_4_ powder materials: (**a**) F0, (**b**) F4, (**c**) F6, (**d**) F8.

To further identify the crystal structure of pristine and Fe-doped NiCo_2_O_4_ powder materials, Rietveld refinement was performed on the collected XRD data, and the corresponding results are presented in Figure 2 and Table 1. All patterns can be well fitted based on the cubic spinel structure with the space group of Fd-3m. The inset shows the crystal structure, where spheres in different colors represent Co, Ni, and O atoms. The experimental diffraction profiles show high consistency with the calculated curves, and the difference curves randomly distribute around the zero line. No obvious impurity diffraction peaks are detected, demonstrating that Fe doping does not alter the dominant crystal structure of NiCo_2_O_4_. According to the refined structural parameters, the lattice constant gradually increases from 8.11391 Å to 8.11783 Å as the Fe doping content rises from F0 to F8, accompanied by an increase in unit cell volume from 534.185 Å^3^ to 534.958 Å^3^. This variation reveals that the incorporation of Fe ions induces slight lattice expansion. When Fe ions substitute for metallic sites within the NiCo_2_O_4_ lattice, the local bond lengths and coordination environments are altered, which is responsible for the increased unit cell parameters. Moreover, all samples exhibit low refinement residuals. The values of R_p_ and R_wp_ range from 6.57% to 7.05% and 8.24% to 9.01%, respectively. The goodness-of-fit factor χ^2^ varies between 1.02 and 1.06, which is close to unity. These satisfactory fitting parameters confirm the excellent agreement between the refinement model and experimental data, as well as the high reliability of the obtained structural parameters. In summary, all Fe-doped NiCo_2_O_4_ powder materials retain a stable spinel phase. The introduction of Fe only slightly modulates the lattice parameters without generating new crystalline phases.

**Figure 3:**
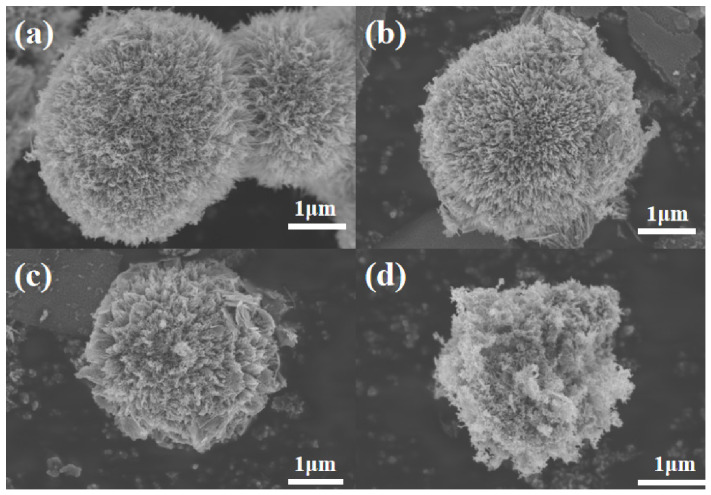
SEM patterns of pristine and Fe-doped NiCo_2_O_4_ powder materials: (**a**) F0, (**b**) F4, (**c**) F6, (**d**) F8.

Figure 3 displays the morphological characteristics of pristine and Fe-doped NiCo_2_O_4_ powder materials. It can be observed that samples F4 (4 at.% Fe) and F6 (6 at.% Fe) exhibit morphologies identical to that of the undoped sample F0 (0 at.% Fe); all samples present urchin-like microspheres self-assembled by nanoneedles with particle sizes ranging from approximately 3 to 5 μm. As the Fe doping concentration increases, the interspace between adjacent nanoneedles gradually expands, accompanied by nanoneedle agglomeration, which enlarges the voids on the microsphere surface. Notably, the inter-needle spacing in sample F6 is significantly larger than that in F4, and the microsphere surface presents more voids, leading to an increased specific surface area. This porous structure provides abundant active sites for multiple reflections and energy dissipation of electromagnetic waves, thereby facilitating electromagnetic wave absorption [23]. As the doping concentration further increases, sample F8 fails to maintain an intact nanosphere structure and transforms into a cluster structure composed of numerous nanosheets, disorderly interwoven and stacked. At Fe doping levels of 4% and 6%, Fe^3+^ ions are uniformly incorporated into the NiCo_2_O_4_ lattice, causing only slight lattice distortion without disrupting the self-assembly behavior, thus preserving the urchin-like microsphere morphology. When the Fe doping level is increased to 8%, the excessive Fe^3+^ exceeds the solid solubility limit of the spinel structure, leading to aggravated lattice distortion and disrupting the oriented growth of nanosheets. Simultaneously, secondary nucleation induced by the excess Fe source, along with increased surface energy, promotes disordered stacking of nanosheets, ultimately forming irregular porous agglomerates.

**Figure 4:**
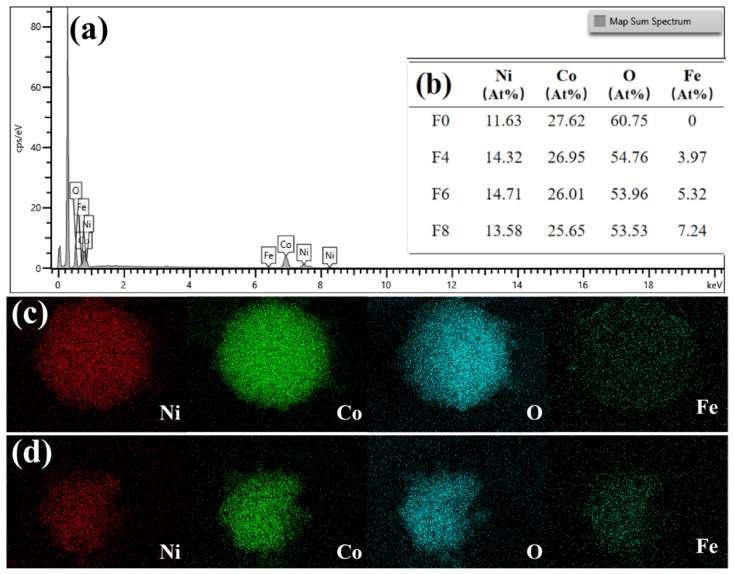
(**a**,**b**) EDS energy spectrum diagram of pristine (F0) and Fe-doped NiCo_2_O_4_ powder materials (F4, F6, F8) and the corresponding component ratios, (**c**,**d**) F6 and F8 elemental mapping.

Energy-dispersive X-ray spectroscopy (EDS) was utilized to investigate the elemental composition and elemental distribution of pristine and Fe-doped NiCo_2_O_4_ powder materials, as illustrated in Figure 4. Figure 4a presents distinct characteristic diffraction peaks assigned to nickel (Ni), cobalt (Co), oxygen (O), and iron (Fe) for sample F6. As shown in Figure 4c,d, the homogeneous distribution of Ni, Co, O, and Fe elements across the entire microstructures confirms the successful incorporation of Fe into the NiCo_2_O_4_ matrix. Quantitative analysis presented in Figure 4b provides further insight into the doping behavior. The atomic percentage of Fe increases progressively with the elevated Fe content in the as-prepared precursor solution, rising from 3.97 at.% for F4 to 5.32 at.% for F6 and further to 7.24 at.% for F8. Correspondingly, the atomic percentages of Ni and Co decrease, while the O content remains relatively stable. These results not only verify the successful elemental doping, but also reveal that the substitutional doping efficiency is governed by the hydrothermal treatment procedure and may be restricted by the number of available suitable lattice sites within the spinel framework.

**Figure 5:**
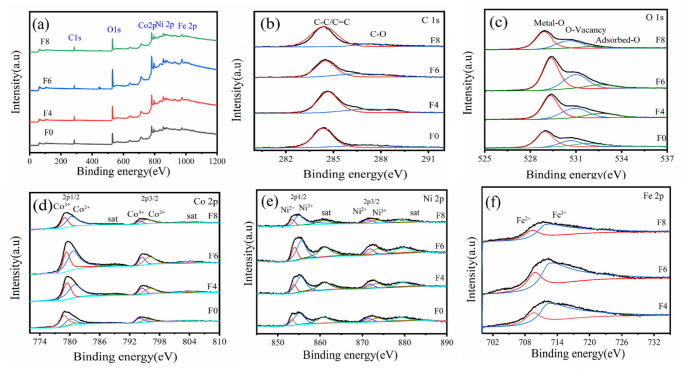
XPS spectra of pristine (F0) and Fe-doped NiCo_2_O_4_ powder materials (F4, F6, F8): (**a**) survey spectrum, (**b**) C 1s, (**c**) O 1s, (**d**) Co 2p, (**e**) Ni 2p, (**f**) Fe 2p.

Figure 5 presents the analysis results of the chemical composition and valence states of pristine and Fe-doped NiCo_2_O_4_ powder materials. The XPS survey spectrum in Figure 5a confirms the presence of C 1s, O 1s, Ni 2p, Co 2p, and Fe 2p in all materials. As shown in Figure 5b, the C 1s spectrum exhibits three distinct characteristic peaks, which are attributed to the C–C (about 284.8 eV) and C–O (about 287.8 eV) functional groups, respectively. In Figure 5c, the O 1s spectrum exhibits peaks at approximately 532.00 eV and 530.70 eV, corresponding to adsorbed oxygen and oxygen vacancies, respectively, while the binding energy at around 529.0 eV is attributed to the metal–oxygen bond. The presence of oxygen vacancies in the sample leads to uneven charge distribution; these defect regions can serve as polarization centers, inducing dipole polarization and defect polarization under electromagnetic field exposure. The high-resolution Co 2p spectrum (Figure 5d) consists of two spin–orbit peaks and two satellite peaks (approximately 788.29 eV and 803.40 eV), indicating the coexistence of Co^2+^ and Co^3+^ [24,25]. Specifically, the peaks at approximately 780.08 eV and 795.42 eV are attributed to Co^2+^, whereas those at about 793.85 eV and 778.78 eV correspond to Co^3+^. The high-resolution 2p spectrum of Ni is shown in Figure 5e, exhibiting two spin–orbit double peaks and two satellite peaks (approximately 861.8 eV and 879.2 eV), indicating the coexistence of Ni^2+^ and Ni^3+^ species. Specifically, the peaks at around 853.32 eV and 871.83 eV correspond to Ni^2+^, while the binding energies at approximately 855.56 eV and 873.56 eV are characteristic of Ni^3+^ [26]. Figure 5f displays the high-resolution Fe 2p spectrum, in which two characteristic peaks are observed: the peak at approximately 709.53 eV is attributed to Fe^2+^, and the peak at approximately 712.24 eV is assigned to Fe^3+^ [27].

In general, an obvious lattice mismatch exists between Co^2+^/Co^3+^ and Fe^3+^ [28]. Meanwhile, the introduction of Fe^3+^ induces abundant intrinsic lattice defects, which generate numerous lattice vacancies and interfacial active sites within the material [29]. Under the excitation of an external electromagnetic field, these lattice defects can induce additional defect dipoles, effectively strengthening the dipole polarization of the system and further accelerating the Debye relaxation process. Meanwhile, electrons tend to undergo energy level transition and charge trapping during migration across defective regions, enabling the controllable regulation of the electrical conductivity of the material [30,31]. Table 2 lists the chemical states of Ni, Co, Fe, and O in all pristine and Fe-doped NiCo_2_O_4_ powder materials, along with their corresponding binding energies (BE) and relative abundances. According to the characterization data in Table 2, with increasing Fe content, the relative area of the oxygen vacancy peak increases from 26.9% (F0) to 31.94% (F4), reaches a maximum of 34.12% (F6), and slightly decreases to 33.86% (F8). This trend confirms that moderate Fe doping significantly promotes the formation of oxygen vacancies, which act as strong polarization centers to enhance dipole relaxation and optimize impedance matching [32]. The slightly reduced vacancy content in F8 sample is likely due to excessive Fe^3+^ substitution partially alleviating charge imbalance. The Fe-doped F6 sample possesses the highest oxygen vacancy concentration. These vacancies act as polarization centers to strengthen dipole relaxation and optimize impedance matching. The Co 2p and Ni 2p spectra reveal that F6 has relatively high contents of Co^2+^ and Ni^2+^, which form a well-balanced mixed-valence system and provide abundant sites for electron hopping, thereby improving conductive loss. The Fe 2p spectra verify the coexistence of Fe^2+^ and Fe^3+^ in all doped samples, and F6 exhibits the highest Fe^2+^/Fe^3+^ ratio of 29.35/70.65, which effectively enhances interfacial polarization. Overall, the maximum oxygen vacancy concentration, balanced Ni/Co valence distribution, and optimal Fe^2+^/Fe^3+^ ratio in the F6 sample synergistically boost dielectric loss and conductive loss, establishing a crucial electronic structural foundation for its superior microwave absorption performance.

**Figure 6:**
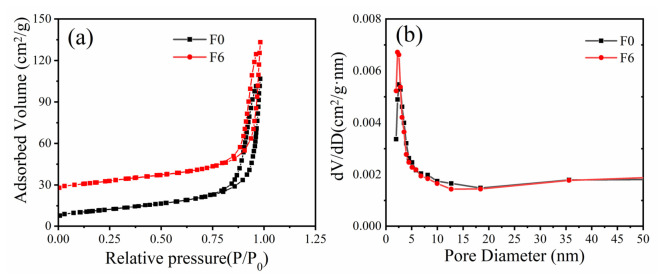
(**a**) N_2_ adsorption/desorption isotherm, (**b**) pore size distribution of pristine (F0) and Fe-doped NiCo_2_O_4_ powder materials (F6).

Figure 6 shows the N_2_ adsorption–desorption isotherms and corresponding BJH pore size distribution curves of pristine NiCo_2_O_4_ (F0) and Fe-doped NiCo_2_O_4_ (F6) samples. Both samples exhibit typical type IV isotherms with distinct H3-type hysteresis loops according to the IUPAC classification, indicating that the materials are dominated by mesoporous structures formed by particle aggregation. The specific surface areas calculated by the Brunauer–Emmett–Teller (BET) method are 40.183 m^2^/g for F0 sample and 46.85 m^2^/g for F6 sample, respectively. The pore size distribution curves reveal that both samples possess mesopores concentrated in the range of 2–10 nm. Fe doping does not significantly alter the pore size distribution, but refines particle size and inhibits agglomeration, thereby increasing the pore number and total pore volume. The higher specific surface area and uniform mesoporous structure of F6 sample provide abundant scattering sites and heterogeneous interfaces for incident electromagnetic waves, which are beneficial to enhancing multiple reflections, interfacial polarization, and optimizing impedance matching.

**Figure 7:**
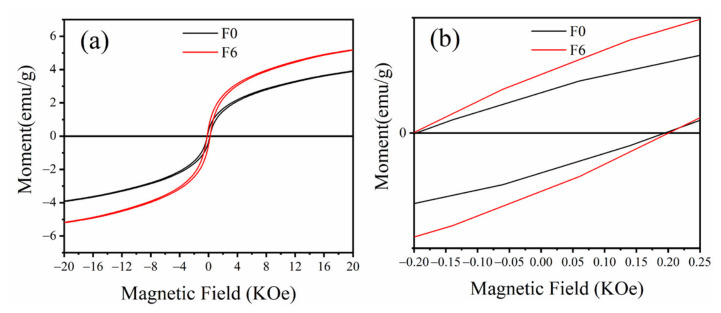
(**a**,**b**) Field-dependent magnetization curve of pristine (F0) and Fe-doped NiCo_2_O_4_ powder materials (F6).

The room-temperature magnetic hysteresis loops of pristine (F0) and Fe-doped NiCo_2_O_4_ powder materials (F6) (Figure 7a) both exhibit typical soft magnetic behavior with negligible remanence and coercivity. The saturation magnetization Mc of pristine NiCo_2_O_4_ (F0) is 3.88 emu/g, while that of Fe-doped NiCo_2_O_4_ powder materials (F6) increases to 5.76 emu/g. This enhancement is attributed to the substitution of Fe^3+^ ions for Ni/Co sites, which introduces higher intrinsic magnetic moments and regulates cation occupancy in the spinel lattice, thereby strengthening the net magnetic coupling of the system. The enlarged view near the origin (Figure 7b) reveals that both samples maintain low coercivity Hc. Specifically, the Hc of F0 sample is 0.194 kOe, while that of F6 sample slightly increases to 0.202 kOe. This variation is directly related to Fe doping-induced grain refinement and increased lattice defects, which act as domain wall pinning centers and enhance the resistance to magnetization reversal.

**Figure 8:**
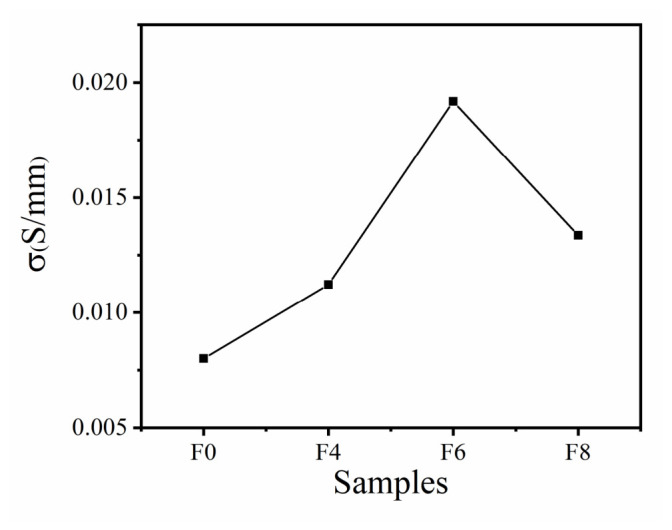
Electrical conductivity of pristine (F0) and Fe-doped NiCo_2_O_4_ powder materials (F4, F6, F8).

The electrical conductivity of pristine (F0) and Fe-doped NiCo_2_O_4_ powder materials (F4, F6, F8) is presented in Figure 8. As the Fe doping content increases from 0 to 6%, the conductivity rises from approximately 0.008 S/mm to a peak of about 0.019 S/mm. This enhancement is attributed to the synergistic effects of mixed-valence Fe ions, introduced oxygen vacancies, and refined grains, which collectively increase the carrier concentration and mobility. When the doping level further increases to 8%, the conductivity decreases to approximately 0.013 S/mm, owing to severe lattice distortion and particle agglomeration induced by excessive Fe doping, which partially disrupts the continuity of the conductive network and increases electron scattering. The peak conductivity of F6 sample maximizes the contribution of conductive loss, which synergistically strengthens with dielectric polarization, serving as one of the key factors supporting its excellent microwave absorption performance.

**Figure 9:**
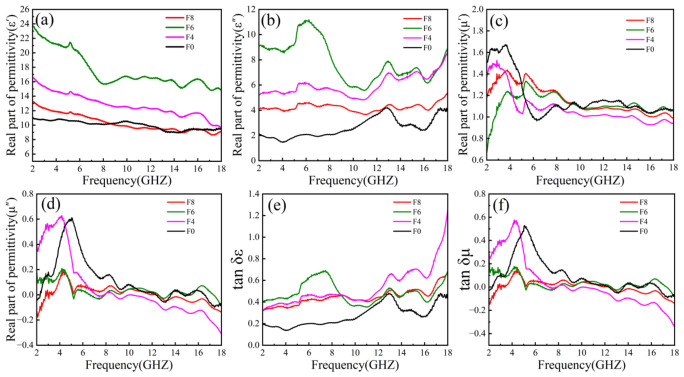
(**a**) Real part of permittivity, (**b**) imaginary part of permittivity, (**c**) real part of permeability, (**d**) imaginary part of permeability, (**e**) dielectric loss tangent, and (**f**) magnetic loss tangent of pristine (F0) and Fe-doped NiCo_2_O_4_ powder materials (F4, F6, F8).

Figure 9 presents the electromagnetic parameters of the pristine and Fe-doped NiCo_2_O_4_ powder materials. The complex permittivity (*ε*_r_ = *ε*′ − j*ε*″) and complex permeability (*μ*_r_ = *μ*′ − j*μ*″) are fundamental electromagnetic descriptors: *ε*′ and *μ*′ quantify electric and magnetic energy storage capacity, respectively; positive *ε*″ and *μ*″ correspond to electric and magnetic energy dissipation, while negative *μ*″ arises from phase lag coupling effects during vector permeability measurement and does not signify energy input to the magnetic field.

As shown in Figure 9, the electromagnetic parameters of the pristine and Fe-doped NiCo_2_O_4_ powder materials exhibit generally consistent trends over the frequency range of 2–18 GHz. Figure 9a and Figure 9b display the frequency spectra of the real part (*ε*′) and imaginary part (*ε*″) of permittivity, respectively. For samples F0, F4, F6, and F8, the *ε*′ values vary within the ranges of 8.98–10.91, 14.73–23.67, 9.45–16.55, and 8.64–13.23, respectively, while the corresponding *ε*″ values lie in the ranges of 1.49–4.17, 5.56–11.17, 4.84–8.53, and 3.89–5.41, respectively. Broad undulations on both permittivity curves stem from overlapping wideband Maxwell–Wagner (M–W) interfacial polarization and defect-induced dipole Debye relaxations [33,34]. M–W polarization dominates charge accumulation at Fe-NiCo_2_O_4_ heterogeneous interfaces and grain boundaries, producing relaxation spanning the full 2–18 GHz range instead of narrow discrete Debye peaks. Compared with pristine NiCo_2_O_4_(F0), the permittivity values of the F4, F6, and F8 samples are enhanced to varying degrees. This phenomenon can be explained by the fact that Fe doping introduces strongly magnetic ions, enhances electron hopping behavior, and induces lattice distortions that create defects. Together with the synergistic effect of multiple polarization mechanisms, these factors result in a significantly higher permittivity than that of pristine NiCo_2_O_4_.

Figure 9c and Figure 9d show the frequency spectra of the real part (*μ*′) and imaginary part (*μ*″) of permeability, respectively. The *μ*′ and *μ*″ values of all samples are relatively small, indicating that the Fe-NiCo_2_O_4_ powder materials possess weak magnetic storage and magnetic loss capabilities [35]. Nevertheless, resonance peaks can still be observed in the *μ*′ and *μ*″ curves, which may be collectively attributed to eddy current loss and natural resonance. Notably, F8 sample delivers negative (*μ*″) at low frequencies (2–4 GHz), a measurement phase-coupling artifact without physical energy amplification.

Figure 9e and Figure 9f plot the frequency dependence of dielectric loss tangent (tan*δ_ε_* = *ε*″/*ε*′) and magnetic loss tangent (tan*δ_μ_* = *μ*″/*μ*′). The tan *δ_ε_* values fluctuate from 0.1 to 1.3, while tan *δ*_μ_ varies between −0.2 and 0.6. Negative tan *δ_μ_* arises from over −90° phase lag between internal magnetic dipoles and the external alternating microwave magnetic field under strong dielectric–magnetic coupling, a common vector resonance phenomenon in porous spinel absorbers, which does not signify energy output from the material. Quantitative statistical averaging over the 2–6 GHz low-frequency region yields clear comparative values: pristine F0 presents average tan *δ_ε_* = 0.22 and average tan *δ_μ_* = 0.15; F4 exhibits average tan *δ_ε_* = 0.48 and average tan *δ_μ_* = 0.31; F6 shows average tan *δ*_ε_ = 0.52 and average tan *δ_μ_* = 0.24; F8 has average tan *δ_ε_* = 0.39 and average tan *δ_μ_* = 0.18. These results confirm that tan *δ_ε_* is persistently larger than tan *δ_μ_* for every sample within 2–6 GHz. Accordingly, dielectric loss acts as the primary electromagnetic wave attenuation channel across the entire 2–18 GHz measurement band. Within low frequencies (2–6 GHz), weak magnetic loss delivers auxiliary synergistic energy dissipation alongside dominant dielectric loss. At high frequencies of 12–18 GHz, tan*δ_ε_* rises continuously while tan*δ_μ_* declines, which further strengthens the dominant position of dielectric loss for microwave energy consumption [36]. Relative to pristine F0, Fe doping significantly boosts tan*δ_ε_* via enhanced Maxwell–Wagner interfacial polarization, defect dipole relaxation and electron-hopping conductive loss; the promotion effect on tan*δ_μ_* is mild, consistent with the weak intrinsic magnetic characteristics of spinel NiCo_2_O_4_ matrix.

According to the Debye relaxation theory, the dielectric properties of a material can be further investigated based on the relationship between *ε*′ and *ε*″, and the expression is as follows:

(1) $${\left(\varepsilon^{\prime }-\frac{\varepsilon_{s}+\varepsilon_{\infty }}{2}\right)}^{2}+{\left(\varepsilon^{\prime \prime }\right)}^{2}={\left(\frac{\varepsilon_{s}-\varepsilon_{\infty }}{2}\right)}^{2}$$

where $\varepsilon_{\infty }$ and $\varepsilon_{s}$ represent the optical dielectric constant and the static dielectric constant, respectively. If the Cole–Cole plot consists of a single semicircle, it corresponds to a single Debye relaxation process [37,38].

**Figure 10:**
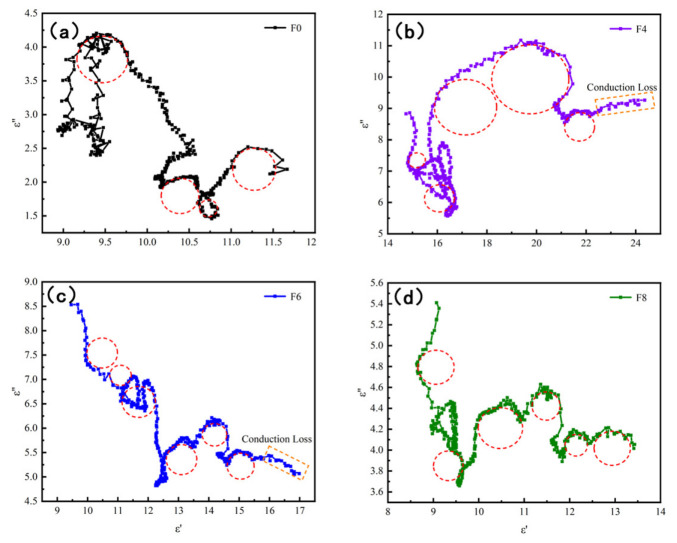
Cole–Cole semicircle plots of (**a**) pristine (F0) and (**b**–**d**) Fe-doped NiCo_2_O_4_ powder materials (F4, F6, F8).

Figure 10 displays the Cole–Cole plots *ε*″ vs. *ε*′ of the pristine and Fe-doped NiCo_2_O_4_ powder materials. Each distorted semicircle corresponds to a dielectric polarization relaxation process. The arc diameter reflects the magnitude of polarization loss, while arc irregularities indicate the distribution of relaxation times arising from heterogeneous polarization environments. The linear trailing segment extending toward the high-frequency side originates from conductive loss; its slope is positively correlated with material conductivity and quantifies the instantaneous energy dissipation capacity of free charge carriers. Collectively, polarization relaxation and conductive loss constitute the two core components of total dielectric loss. For undoped F0 (Figure 10a), a single nearly intact semicircle dominates the profile, demonstrating that polarization relaxation serves as the primary attenuation channel while conductive loss contributes negligibly. After 4 at.% Fe doping (F4, Figure 10b), two prominent large distorted semicircles emerge, revealing that Fe substitution introduces abundant heterogeneous interfaces and lattice defects to remarkably strengthen Maxwell–Wagner interfacial polarization and defect-induced dipole polarization. Meanwhile, the steeper slope of the high-frequency linear tail indicates a noticeable elevation in conductive loss. For 6.4 at.% Fe F6 (Figure 10c), distinct semicircular arcs coexist with moderate linear trailing segments. Compared with F4, its conductive loss slightly decreases while the area of relaxation arcs partially recovers, indicating a well-balanced synergistic effect between polarization relaxation and conductive loss. In the case of 8 at.% Fe-doped NiCo_2_O_4_ powder materials (Figure 10d), the linear conductive tail nearly disappears, and polarization relaxation becomes the dominant dielectric loss pathway. In summary, rational regulation of Fe doping content can coordinate the competitive contributions of conductive loss and polarization relaxation, and the F6 sample achieves the optimal synergistic matching between these two attenuation mechanisms.

According to the transmission line theory of electromagnetic waves, the reflection loss (*RL*) of ferrite-based absorbing materials is calculated as follows:

(2) $$RL=20lg\left|\frac{Z_{in}-Z_{0}}{Z_{in}+Z_{0}}\right|$$

(3) $$Z_{0}=\sqrt{\frac{\mu_{0}}{\varepsilon_{0}}}$$

(4) $$Z_{in}=Z_{0}\sqrt{\frac{\mu_{r}}{\varepsilon_{r}}}\tan h\left[j\frac{2\pi fd}{c}\sqrt{\mu_{r}\varepsilon_{r}}\right]$$

where *Z*_0_ and *Z_in_* represent the input impedances of free space and the material, respectively; *f*, *d*, and *c* correspond to the frequency, thickness, and speed of light, respectively; *μ*_0_ and *ε*_0_ are the vacuum permeability and permittivity, respectively. An *RL* value of less than −10 dB indicates that 90% of the incident energy is absorbed.

**Figure 11:**
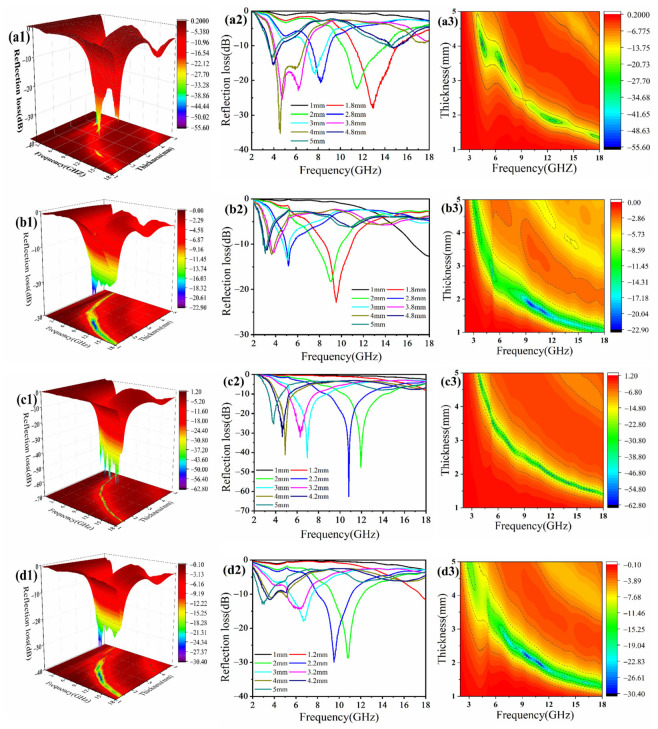
Reflection loss (RL) of pristine (F0) and Fe-doped NiCo_2_O_4_ powder materials (F4, F6, F8) (2–18 GHz, thickness: 1.0–5.0 mm): (**a1**–**d1**) 3D RL surface plots; (**a2**–**d2**) 2D RL curves with variable thicknesses; (**a3**–**d3**) 2D RL contour maps.

Figure 11a–d show the reflection loss (*RL*) curves of pristine and Fe-doped NiCo_2_O_4_ powder materials. The microwave absorption performance of the pristine NiCo_2_O_4_ (F0) sample is presented in Figure 11(a1–a3). The minimum reflection loss (*RL*_min_) of the F0 sample is approximately −50.69 dB at 11.98 GHz, with a corresponding matching thickness of 4.0 mm, and its effective absorption bandwidth (EAB) reaches 3.1 GHz. As shown in Figure 11(b1–b3), F4 sample achieves a minimum reflection loss of –22.87 dB at a matching thickness of 1.8 mm and a frequency of 9.67 GHz. As displayed in Figure 11(c1–c3), F6 sample exhibits a minimum reflection loss of –62.7 dB at a matching thickness of 2.2 mm and a frequency of 10.19 GHz, with a corresponding effective absorption bandwidth (*RL* < –10 dB) of 4.6 GHz. Upon further increasing the Fe doping level to 8 at.%, as shown in Figure 11(d1–d3), F8 sample attains a minimum reflection loss of –29.85 dB at a matching thickness of 2.2 mm and a frequency of 9.45 GHz, along with an effective absorption bandwidth (*RL* < –10 dB) of 2.9 GHz. F4 and F8 samples exhibit relatively poor reflection loss performance, primarily due to insufficient doping concentration or excessive doping, which hinders the construction of an efficient electromagnetic loss network [39]. At a low Fe doping level (4 at.%), the introduction of foreign ions is insufficient to disrupt the thermodynamic equilibrium of the original lattice, making it difficult to induce sufficient oxygen vacancies or metal cation defects within the system. Moreover, the single-phase doped solid solution structure cannot form abundant phase boundaries, resulting in very limited heterojunction interfaces. Conversely, when the Fe doping level is too high (8 at.%), exceeding the solid solubility limit of the matrix material, the excess Fe ions not only cause severe lattice distortion or even collapse, but also promote Fe segregation and the in situ precipitation of a secondary phase (such as Fe_2_O_3_ or Fe_3_O_4_) on the surface. Although such excessive phase separation can locally construct heterojunctions, it is accompanied by coarsening and agglomeration of the nanostructures, which instead masks the abundant active defect sites that would otherwise be exposed. When the doping level is increased to that of 6 at.%, the introduction of Fe^3+^ achieves a favorable synergistic balance between conductive loss and magnetic loss, thereby enabling electromagnetic waves to efficiently enter the material interior and be effectively attenuated [40]. Table 3 compares the microwave absorption performance of the Fe-NiCo_2_O_4_ powder materials prepared in this work with previously reported NiCo_2_O_4_-based absorbers. At a matching thickness of 2.2 mm, the as-prepared sample exhibits a minimum reflection loss RL of −62.7 dB and an effective absorption bandwidth (EAB) of 4.6 GHz. Compared with pure NiCo_2_O_4_, NiCo_2_O_4_-rGO, NiCo_2_O_4_/ZnO, and other reported Fe-doped NiCo_2_O_4_ materials, the Fe-NiCo_2_O_4_ (F6, 6 at.%) shows both stronger attenuation capability and broader absorption bandwidth, outperforming most single-modified or binary composite systems.

**Figure 12:**
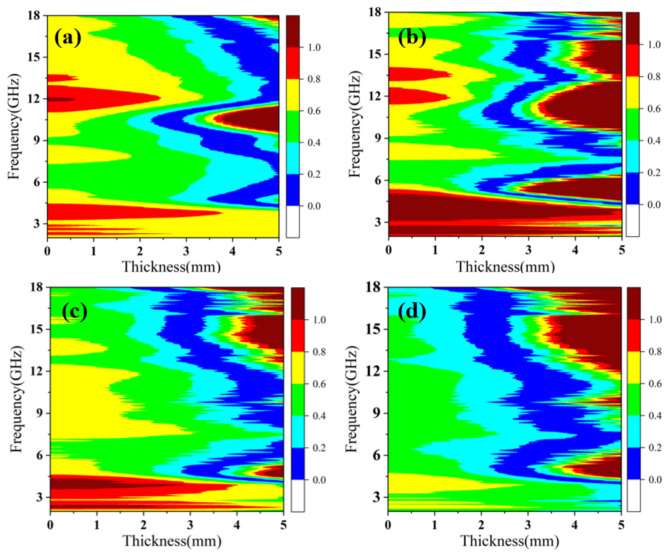
Delta (Δ) values of pristine and Fe-doped NiCo_2_O_4_ powder materials; (**a**) F0, (**b**) F4, (**c**) F6 and (**d**) F8.

To further quantitatively evaluate the impedance matching characteristics of the Fe-doped NiCo_2_O_4_ powder materials, the impedance matching coefficient (Δ value) was introduced, as shown in Figure 12. The Δ value is a dimensionless parameter calculated based on the complex permittivity and complex permeability of the material, serving to characterize the impedance matching degree between the absorbing material and free space. Generally, a smaller Δ value indicates closer proximity between the impedance of the material and that of free space, facilitating the reflectionless entry of electromagnetic waves into the material. In this work, Δ ≤ 0.4 was adopted as the critical threshold to define the effective impedance matching region. As illustrated by the three-dimensional impedance matching coefficient distribution maps, the horizontal axis represents the matching thickness, while the vertical axis denotes the electromagnetic wave frequency. The color gradient from red to blue signifies a decrease in the Δ value. In stark contrast to the doped samples, the map for the F0 sample (if shown) is predominantly characterized by warm colors (red and yellow), indicating severe impedance mismatch across the entire frequency and thickness range, with almost no effective matching region observed. As illustrated by the three-dimensional impedance matching coefficient distribution maps for F4, F6, and F8, it can be observed that the maps of these samples exhibit extensive regions of low Δ values (blue and green), particularly achieving broadband impedance matching at relatively thin matching thicknesses. Statistical calculations reveal that the area ratios of the effective matching region (Δ ≤ 0.4) for samples F4, F6, and F8 are 28%, 58%, and 30%, respectively. Notably, the high proportion of 58% for sample F6 implies excellent impedance matching characteristics across broad frequency and thickness ranges. This extensive low-Δ region directly corresponds to a wider effective absorption bandwidth and greater flexibility in coating thickness design. This demonstrates that the incorporation of an appropriate amount of Fe (6 at.% doping level) significantly optimizes the electromagnetic parameters of the material, allowing incident electromagnetic waves to penetrate into the absorber to the greatest extent. Consequently, this establishes a prerequisite for achieving high-efficiency broadband electromagnetic wave absorption.

Owing to the ionic radius of Fe^3+^ being similar to that of Co^3+^, Fe^3+^ preferentially substitutes for Co^3+^ in NiCo_2_O_4_, forming a NiFe_x_Co_2−x_O_4_ solid solution. This substitution process breaks the symmetry of the original lattice, induces lattice distortion, and generates defects such as oxygen vacancies and cation vacancies, thereby activating defect polarization relaxation and effectively enhancing the dielectric loss capability of the material. Meanwhile, Fe doping promotes the coexistence of multiple valence states, including Fe^2+^/Fe^3+^, Co^2+^/Co^3+^, and Ni^2+^/Ni^3+^, within the system. Under an external alternating electromagnetic field, electron hopping migration occurs among these multivalent metal ions, which not only increases the electrical conductivity of the material but also enhances the conductive loss. Furthermore, Fe doping effectively tunes the complex permittivity and complex permeability of the material, optimizes the matching of electromagnetic parameters, reduces the reflectivity of electromagnetic waves at the material surface, and allows more electromagnetic waves to penetrate into the material interior [49]. Incident electromagnetic waves undergo multiple reflections and scatterings within the porous structure of Fe-doped NiCo_2_O_4_, significantly prolonging the propagation path, thereby further strengthening the coupling between dielectric loss and magnetic loss. The synergistic effect of the above multiple loss mechanisms ultimately achieves efficient electromagnetic wave absorption and energy dissipation.

## 4. Conclusions

Herein, pristine and Fe-doped NiCo_2_O_4_ powder materials with doping contents of 4%, 6% and 8% were synthesized via a hydrothermal–calcination route, and their microstructure, electronic state, electrical/magnetic properties and microwave absorption performance were systematically studied. The pristine NiCo_2_O_4_ (F0) delivers a minimum reflection loss of −50.69 dB at a matching thickness of 4.0 mm and a frequency of 11.98 GHz, with an effective absorption bandwidth (*RL* < −10 dB) of 3.1 GHz. In comparison, the Fe-doped samples F4, F6 and F8 exhibit minimum reflection loss values of −22.87 dB, −62.7 dB and −29.85 dB, and their effective absorption bandwidths are relatively narrow, 4.6 GHz and 2.9 GHz, respectively. The proportions of regions with favorable impedance matching (Δ < 0.4) for the three samples are 28%, 58% and 30% in sequence. The F6 sample with 6 at.% Fe doping shows the best comprehensive performance, which is attributed to its well-developed porous structure, abundant oxygen vacancies, and balanced Ni/Co/Fe mixed-valence states. These features endow F6 with optimized conductivity and magnetic properties, achieving strong synergism of dielectric, conductive and magnetic losses. In comparison, low doping in F4 cannot form effective loss pathways, while excessive doping in F8 causes lattice distortion and deteriorated defect distribution, both leading to weakened absorption ability. This work clarifies the structure–performance relationship of Fe-doped spinel absorbers and provides a reliable route for designing high-performance microwave-absorbing materials.

## Figures and Tables

**Table 1:** Detailed refinement result of XRD profile for all samples of pristine and Fe-doped NiCo_2_O_4_ powder materials.

Fe Content (x)	Lattice Parameter (Å)		Fitted Goodness		
		v	R_p_	R_wp_	χ^2^
F0	a = b = c = 8.11391	534.185	6.567	8.241	1.02
F4	a = b = c = 8.11469	534.338	6.609	8.401	1.03
F6	a = b = c = 8.11579	534.555	6.730	8.606	1.06
F8	a = b = c = 8.11783	534.958	7.045	9.014	1.04

**Table 2:** The proportions of different valence states of Co, as well as different types of oxygen species in the pristine and Fe-doped NiCo_2_O_4_ powder materials, were calculated and fitted by XPS spectroscopy.

Samples		F0		F4		F6		F8	
		BE (eV)	% Area	BE (eV)	% Area	BE (eV)	% Area	BE (eV)	% Area
**O**	Metal-O	528.9	51.98	529.32	51.04	529.36	54.69	529.0	54.32
	O-Vacancy	530.64	26.9	530.93	31.94	530.99	34.12	530.75	33.86
	Adsorbed-O	531.75	21.12	532.58	17.03	532.57	11.19	532.27	11.83
Co	**Co^2+^**	795.42	11.16	795.98	12.68	795.91	13.42	795.23	15.8
		780.08	31.72	780.88	39.25	780.49	42.79	780.23	47.8
	**Co^3+^**	793.85	11.46	794.37	8.37	794.35	10.12	793.84	7.67
		778.78	37.23	779.34	32.36	779.20	25.45	778.85	22.15
	**Satellite**	803.40	4.03	803.74	3.36	803.57	4.65	803.54	4.13
		788.29	5.41	789.29	3.98	788.66	3.57	787.97	2.46
**Ni**	Ni^2+^	853.32	18.45	853.82	17.55	853.96	20.87	853.37	9.22
		871.83	6.72	871.65	5.94	871.83	6.66	871.34	11.1
	Ni^3+^	855.56	27.38	855.38	28.45	855.56	25.31	854.94	36.87
		873.56	13.54	873.32	14.03	873.56	12.84	873.33	13.26
	**Satellite**	861.09	22.42	861.08	23.17	861.09	23.2	860.97	17.61
		879.61	11.48	879.49	10.87	879.61	11.13	879.24	11.93
**Fe**	Fe^2+^	-	-	709.53	25.36	709.8	29.35	709.27	26.7
	Fe^2+^	-	-	712.24	74.64	712.47	70.65	711.83	73.3

**Table 3:** Microwave absorption performance of the NiCo_2_O_4_ materials.

Material	RL (dB)	EAB (GHZ)	Thickness (mm)	References
Fe-NiCo_2_O_4_(6 at.%)	−62.7	4.6	2.2	This work
NiCo_2_O_4_	−38.5	4.2	1.8	[41]
NiCo_2_O_4_-rGO	−43.5	4.2	2.5	[42]
NiCo_2_O_4_/ZnO	−51.4	4.5	2.2	[43]
NiCo_2_O_4_ NSs/Ti_3_C_2_Tₓ	−68.0	3.6	1.7	[44]
NiCo_2_O_4_/MXene	−72.3	3.6	1.7	[45]
Fe-doped NiCo_2_O_4_	−48.9	4.2	1.5	[46]
C-NiCo_2_O_4_	−52.7	5.2	1.9	[47]
Fe_3_O_4_@NiCo_2_O_4_/PANI/NRGO	50.9	4.2	3.0	[48]

## Data Availability

The original contributions presented in the study are included in the article, and further inquiries can be directed to the corresponding author.
